# Editorial: Databases and Nutrition

**DOI:** 10.3389/fnut.2022.853600

**Published:** 2022-03-18

**Authors:** Alessandra Durazzo, Massimo Lucarini

**Affiliations:** CREA-Research Centre for Food and Nutrition, Rome, Italy

**Keywords:** food composition databases, dedicated databases, harmonization and standardization procedures, ontology, matching process, coding procedures, data traceability, interoperability

## Introduction

Studies examining the relationship between diet and health have led to a growing interest in all the biologically active components found alongside the nutrients in foods. In addition to nutritional function, these components of the diet have potentially beneficial properties: this has led to an increase in the perception of foods as functional and nutraceutical. At the same time new nutrient characteristics are emerging and the boundary between nutrients and bioactive compounds is being explored. The need to update food composition databases and dedicated databases is essential for providing information on the dietary factors contributing to chronic diseases and managing health conditions.

Food, nutrition, and health issues are strictly linked. The recent short survey of Nordhagen et al. (1) examines and highlights a set of specific causal pathways through which food safety and nutrition are interlinked across health and physiology, consumer behavior, supply chains and markets, and policy and regulation. The same authors concluded that integrating nutrition and food safety in food systems policy and programming throughout food supply chains, food environments, consumers, and food system drivers represents a new direction.

The book by Clavier and De Oliveira (2) analyzes the communication strategies of actors and the dissemination and use of information related to both food for health and health through food, considering nutrition from the point of view of public policies, educational organizations, preventive measures, consumers, and patients. For instance, the role of labeling in a sustainable food perspective is discussed as well as the social appropriation of “Diet and Health” and personalized digital tools.

The study of Siegrist et al. (3) examined the effects of psychological traits and nutrition knowledge on perceived risks related to food and nutrition: nutrition knowledge results in greater concern about the food risks that people should be most worried about. The same authors suggested that increasing people's diet-related health consciousness and nutrition knowledge may enhance people's perceptions of lifestyle risks. It is well-known that one of the major obstacles to improving health through diet and lifestyle changes is that they are difficult to implement and perhaps even more difficult to sustain in the long-term. There is now mounting evidence that one way to improve health outcomes through diet and lifestyle change is to increase health consciousness and nutrition knowledge.

In this editorial, we provide a brief overview of the field, followed by a discussion of the research articles published in this special issue on the Research Topic “Databases and Nutrition,” which highlights advances in our knowledge of the intersection between food, nutrition, health, and databases.

## Literature Quantitative Research Analysis: A Shot

To provide a brief shot of this research history and the current status of the field surrounding our Research Topic, we conducted a literature search. The high level results are reported in this section. We then discuss the articles published in this Research Topic on “Databases and Nutrition” in the subsequent section.

On 20 November 2021, the Scopus database was used to carry out a search to retrieve publications that referred to databases, food, nutrition, and health relationship.

The search string: “Database^*^” AND “Food^*^” AND “Nutrition^*^” AND “Health^*^” was used to extract bibliometric data from the Scopus online database (https://www.scopus.com/home.uri, accessed on 20 November 2021) and bibliographic data, i.e., publication year, publication count, document type, countries/territories of origin, institutions, were recorded.

The functions of the Scopus web online platform named “Analyze” and “Create Citation Report” were utilized for carrying out basic analyses.

**Figure 1:**
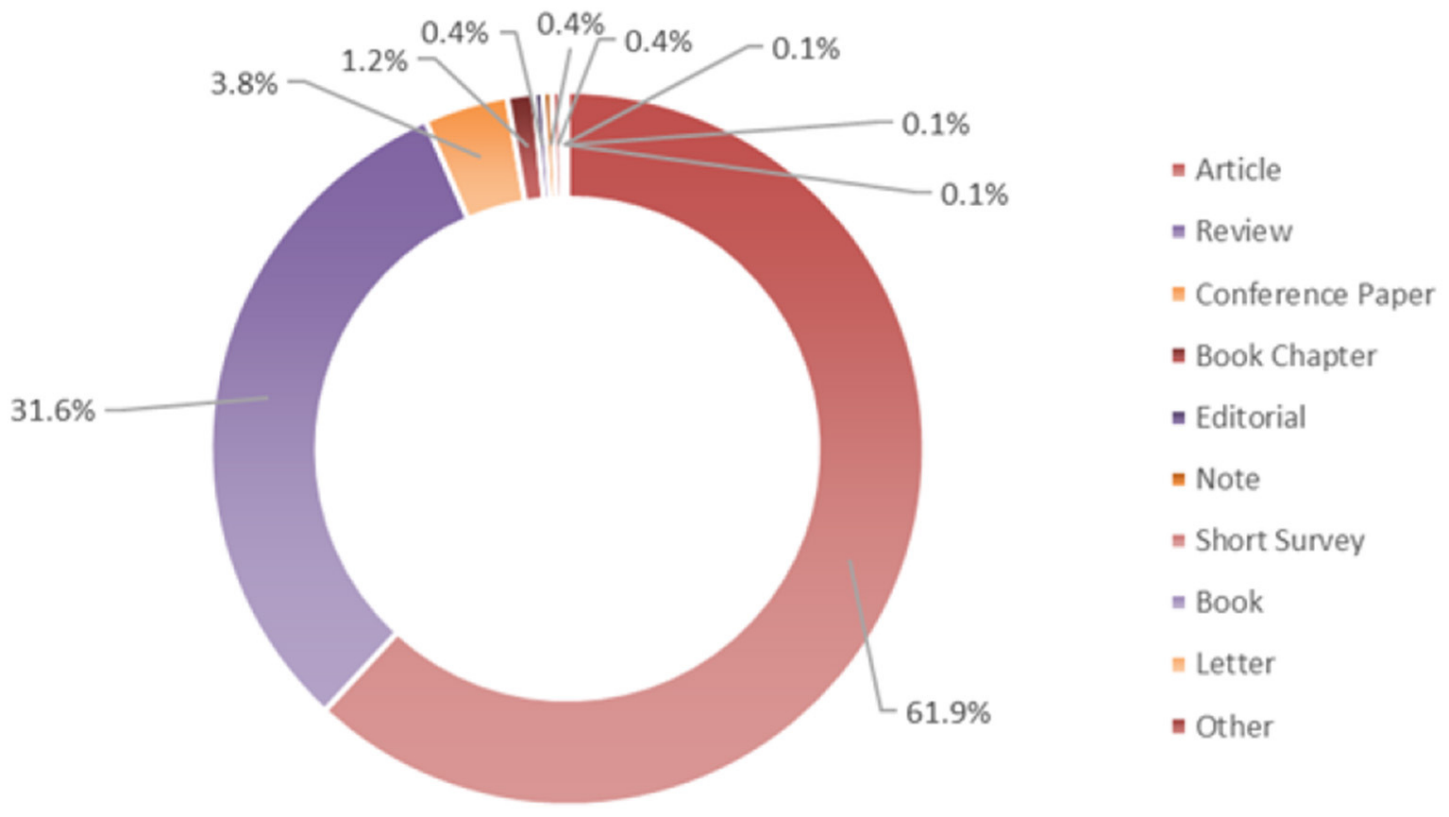
Distribution of documents by type. Based on data from Scopus.

The search returned 3,213 documents covering the period 1943–2022, and mainly the subject areas of *Medicine, Nursing*, and *Agricultural and Biological Sciences*. The papers are distributed per typology as reported in Figure 1 and include mainly “Articles” for the 61.9 %, followed by “Reviews” 31.6%, then “Conference papers” (3.8%), “Book chapters” (1.2%), and so on.

The oldest document was published by Mead, (4) in 1943 in the journal *Psychological Bulletin* and it is entitled “The Committee on Food Habits.” The most cited document (1,770 times) is by Block et al. (5) on the development of a data-based approach to diet questionnaire design and testing.

The most recent review is a comprehensive study of history, phytochemistry, experimental pharmacology, and clinical uses of honey with special reference to Unani medicine (6).

Among the most current reviews published in 2022, it is worth mentioning the paper published by Larrick et al. (7) in the *Journal of Food Composition and Analysis* as an update on “A Partnership for Public Health: USDA Global Branded Food Products Database” with the goal of improving public health and sharing open data by expanding and enhancing the USDA National Nutrient Database (USDA FoodData Central) with nutrient composition and ingredient information on branded and private label foods to better reflect the food supply.

The most recent “article” is focused on the evaluation of micronutrient composition, antioxidant properties, and mineral safety index of selected Nigerian cooked foods (8).

A modern application is given by the USDA on the use of the Database of Flavonoid Values for USDA Food Codes 2007–2010 in assessing intake differences between the Healthy Aging in Neighborhoods of Diversity across the Life Span (HANDLS) study and What We Eat in America (WWEIA), NHANES (9).

Within the category “Book” the following products were reported: (i) metabolomics in food and nutrition (10); a roadmap to 2050 of science and technology on public health in China (11); overview, funding issues, and trends of U.S. Nutrition and agricultural research (12).

The most cited Editorial (91 times) is entitled “Environmental impacts of food consumption and nutrition: where are we and what is next?” published by Nemecek et al. (13) in 2016 on *International Journal of Life Cycle Assessment*.

**Figure 2:**
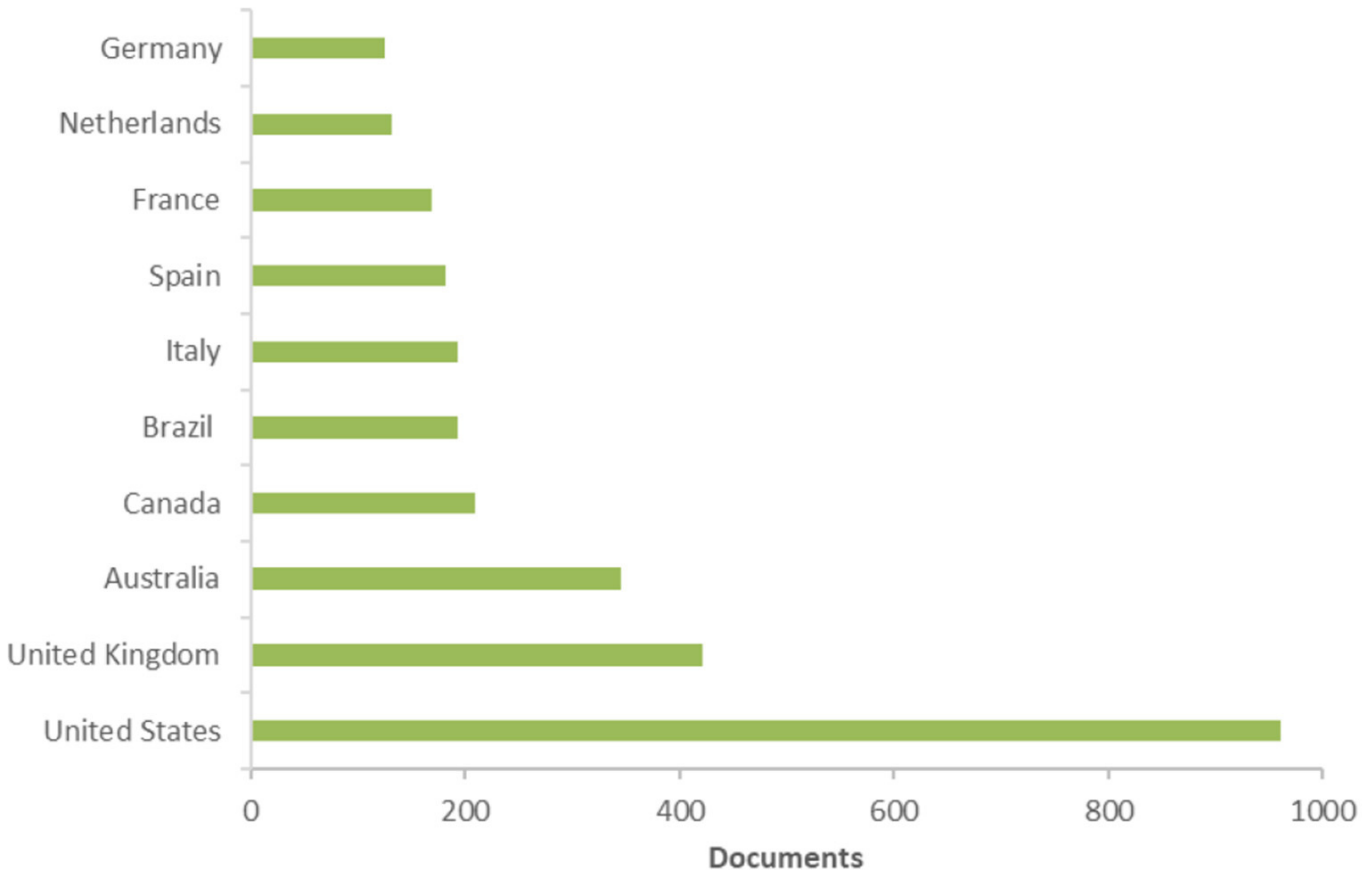
Most productive countries/territories. Based on data from Scopus.

Figure 2 reports, respectively, the most productive countries/territories: United States (*n* = 960) was the most productive country, followed by the United Kingdom (*n* = 422) and Australia (*n* = 345).

For the United States, one short survey addressing the epidemiological evidence on flavonols, flavones, flavanones, and human health is reported (14).

For the United Kingdom, the most cited review discussed the implications of nutritional antioxidants of plant polyphenols in cancer and heart disease (15), whereas the most recent review was focused on the impact of the 2008 Great Recession on dietary intake (16).

The most cited article (541 items) is a document published by Kroes et al. (17) in 2004 in the journal *Food and Chemical Toxicology* as guidance for the application of substances and their presence at low levels in the diet, with a focus on structure-based thresholds of toxicological concern.

For Australia, the most cited documents are as follows: (i) the WHO Health Promoting School framework for improving the health and well-being of students and their academic achievement (18); definition of the Mediterranean diet (19); diet quality (20); the relationship between nutrition knowledge and dietary intake (21).

**Figure 3:**
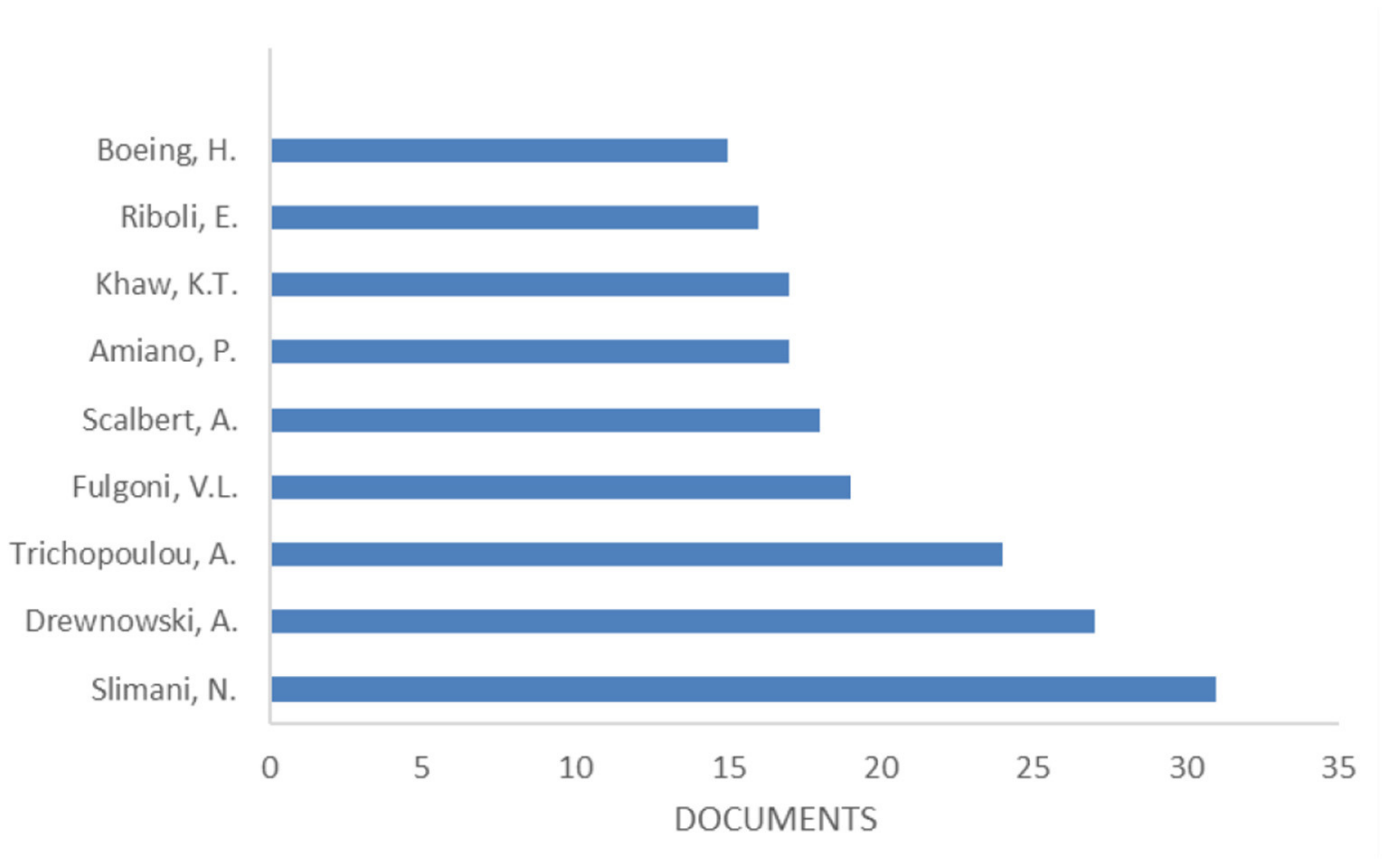
Most productive Authors. Based on data from Scopus.

Figure 3 reports the most productive authors with Slimani, N., publishing 31 documents, having the most. Her most cited paper (268 times) described a first attempt to standardize nutrient databases across the 10 European countries participating in the EPIC study: the EPIC nutrient database project (22).

Narrowing the search toward the interface database/nutrients/bioactive components using the search string: “Database^*^” AND “Food^*^” AND “Nutrition^*^” AND “Health^*^” AND “Composition^*^” OR “Nutrient^*^” OR “bioactive compound^*^” OR “bioactive component^*^” OR “bioactive molecule^*^” OR “dietary supplement^*^” the search returned 1,469 documents.

The “full records and cited references” were exported to VOSviewer software for further bibliometric analyses and additional processing, (version 1.6.16, www.vosviewer.com) (23–25).

**Figure 4:**
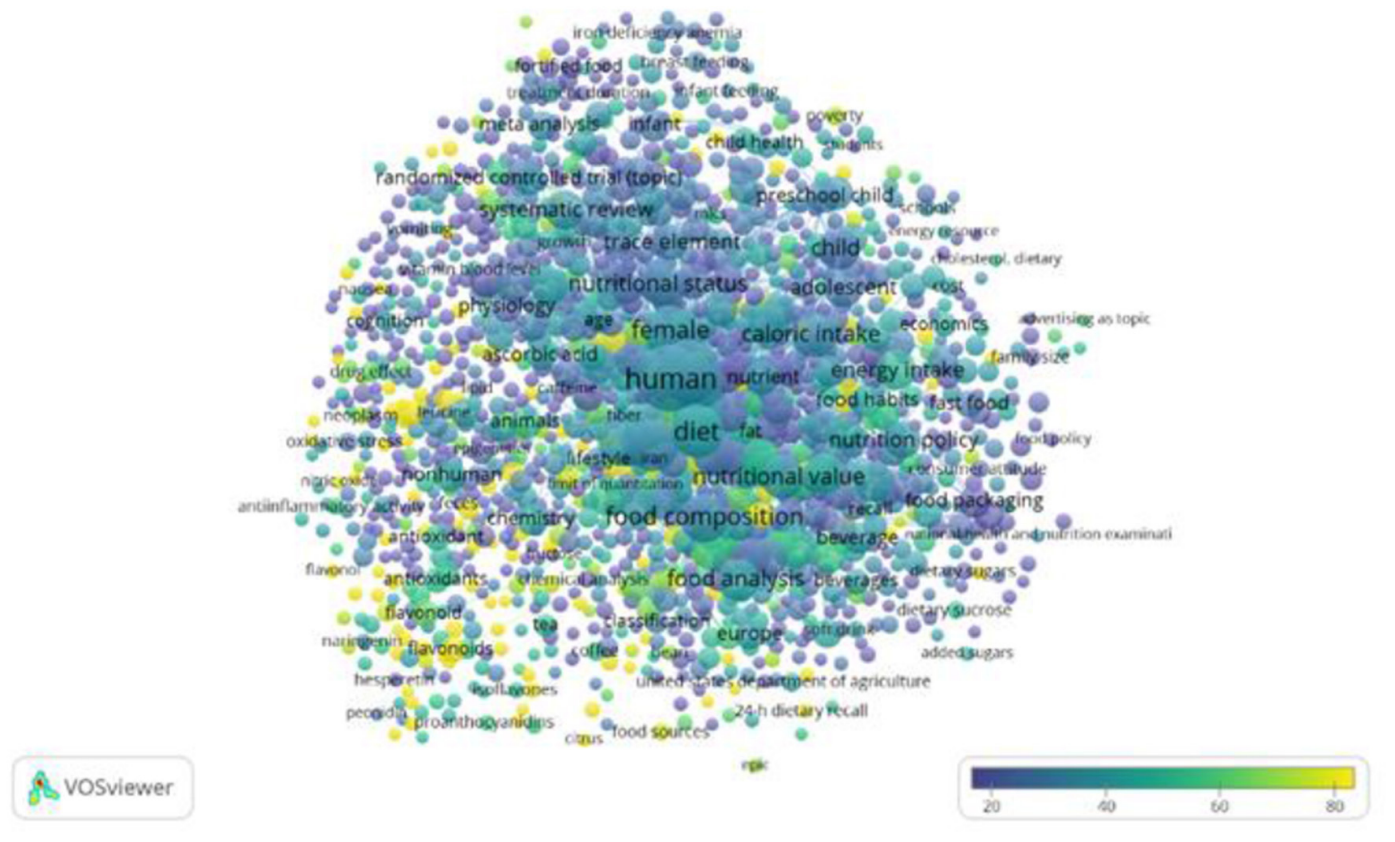
Term map for search database, food, nutrition, health, composition, nutrient, bioactive compound, bioactive component, bioactive molecule, dietary supplement relationships. Bubble color represents the citations per publication (CPP). Two bubbles are closer to each other if the terms co-appeared more frequently. Based on data from Scopus and elaborated by VOSviewer software.

Out of the 9,237 keywords, 1,376 meet the selected threshold, whereas 4 of them were manually excluded, and are represented in a term map (Figure 4).

The most recurring terms are human/s, diet, female, nutrition, male, adult, food composition, dietary intake, food intake, caloric intake, nutritional value, nutritional assessment, food analysis, child, and nutritional status.

This bibliometric analysis demonstrates a growing interest in the development and refinement of databases for food, nutrients, and bioactive molecules. There is a clear and growing need to define the composition of the foods we eat to be able to increase our understanding of their effects on health outcomes. A growing interest in capturing the content of foods in accurate databases, from regional foods to branded products, was observed across the globe.

## Exploring the Research Topic

In the collection of articles under the Research Topic “Databases and Nutrition,” a total of 19 articles are published, mainly focusing on presenting new databases and/or updates for existing databases, with particular attention to their uses and applications.

It is worth mentioning the work of Samaniego-Vaesken et al. which presented an updated database and trends of declared low- and no-calorie sweeteners from foods and beverages marketed in Spain. A total of 1,238 products were identified and the major groups were sugar and sweets (24%), non-alcoholic beverages (21%), cereals and grains (19%), and milk and dairy products (14%) accounting for >70% of total products. The authors also found that the most declared products were low- and no-calorie sweeteners were sorbitol (19.5%), sucralose (19.5%), and acesulfame K (19.2%) (Samaniego-Vaesken et al.). In a brief research report, Hafner and Pravst showed a sharp rise in the use of low- and no-calorie sweeteners in non-alcoholic beverages in Slovenia as an update based on 2020 data (Hafner and Pravst). Restrepo et al. presented a multi-dimensional dataset of open data and satellite images for the characterization of food security and nutrition. An example of database creation is given also by Palmer et al. on the development of a vitamin K database for commercially available food in Australia.

Poulain et al. presented the Malaysian Food Barometer Open Database for studying the modernization of Malaysian food patterns and their economic and health consequences.

Among discussions of the applications and uses of a database, the use of branded food composition databases is presented as a tool to support nutrition research and monitoring of the food supply, with insights from the Slovenian Composition and Labeling Information System (CLAS) (Pravst et al.) as well as for the exploitation of food fortification practices, throughout a case study on vitamin D in the Slovenian food supply (Krušič et al.). Moreover, Hafner et al. verified the use of food labeling data for compiling branded food databases throughout a case study of sugars in beverages.

In the collection, some articles were focused on the relationship between diet quality and health. Duan et al. reported the design, implementation, and major findings of the Chinese Adolescent Cohort Study. Results from the Chinese Adolescent Cohort Study baseline and the first follow-up data suggested that higher protein intake among girls and unhealthy eating habits among children might increase the risk for childhood obesity. Moreover, the same authors marked higher intakes of grain and meat and lower overall diet quality and intakes of dietary fiber and tuber might be associated with advanced pubertal development (Duan et al.). Ma et al. in a cross-sectional study of the US Population showed a saturation value association between Body Mass Index and Bone Mineral Density for people over 50 years old. The authors suggested how keeping the Body Mass Index in the slightly overweight value (around 26 kg/m^2^) might reduce other adverse effects while obtaining optimal Bone Mineral Density in this age group (Ma et al.).

Shamah-Levy et al. analyze malnutrition, being overweight, and having obesity in children, teenagers, and adults through the National Health and Nutrition Surveys information available from public databases in Mexico from 2006 to 2020.

Some examples of dietary strategies are here presented. Neill et al. showed how vitamin D biofortification of pork may offer a food-based strategy to increase vitamin D intake in the UK Population. O'Connor et al. showed how the heterogeneity in meat food groups can meaningfully alter population-level intake estimates of red meat and poultry.

Ortenzi and Beal identified the top food sources of priority micronutrients, among minimally processed foods for the complementary feeding of children (6–23 months) in South and Southeast Asia throughout an aggregated regional food composition database for South and Southeast Asia. de Amorim et al. showed the use of databases to evaluate the prevalence of hunger among adolescents in Brazil. Meanwhile, the work of Liechti et al. proposes a multicriteria approach to select the best representative products from the market base for future reformulation by going beyond nutrition and composition information on packaging. Tseng et al. described a new, open-source ingredient list search method and applied this method to describe the presence of sensory-related industrial additives in US packaged foods. Mariscal-Arcas et al. described the evolution of nutritional habit behaviors of a Spanish population confined through social media. On the other hand, Timotijevic et al. showed the developing responsible governance for a food and nutrition e-infrastructure, throughout the case study of the determinants and intake data platform.

## Conclusion

This editorial has provided a general bibliographic overview of the Research Topic “Databases and Nutrition” and discusses the papers published in this special issue, including current advances in the development of databases needed for research on the relationships between food, nutrition, and health. It is important to continuously update and implement databases in a standardized and harmonized manner between various organizations and countries, in order to have resources that are more representative and that reflect changes in food environments and markets in the context of evolving consumer dietary choices and habits. New and alternative food sources, and at the same time new descriptors and markers, need to embrace different areas in the perspective of interoperability between databases. Under the One Health approach for food safety, food security, and sustainable food production (26), linking bioresources repositories (i.e., cultivar, alternative sources, etc.) to data describing the chemistry and quality of food products (i.e., food composition data, bioactive components, bioactivity, beneficial properties, etc.) represents a new frontier.

## Author Contributions

AD and ML have made a substantial, direct, and intellectual contribution to the work and approved it for publication.

## Conflict of Interest

The authors declare that the research was conducted in the absence of any commercial or financial relationships that could be construed as a potential conflict of interest.

## Publisher's Note

All claims expressed in this article are solely those of the authors and do not necessarily represent those of their affiliated organizations, or those of the publisher, the editors and the reviewers. Any product that may be evaluated in this article, or claim that may be made by its manufacturer, is not guaranteed or endorsed by the publisher.
